# Sexual stimulatory effects of *Mucuna pruriens* in rodents: An experiment following Ayurvedic perspective

**DOI:** 10.1016/j.jaim.2025.101130

**Published:** 2025-06-09

**Authors:** Muralikrishnan Dhanasekaran, Binu Tharakan, Jeyaram Bharathi Jeyabalan, Suhrud Pathak, Keyi Liu, Rishi Nadar, Timothy Moore

**Affiliations:** a Department of Drug Discovery and Development, Harrison College of Pharmacy, Auburn University, Auburn, AL, 36849, USA; b Department of Surgery, Morehouse School of Medicine, Atlanta, GA, 30310, USA; c Department of Pharmacology, JSS College of Pharmacy, JSS Academy of Higher Education & Research, Nilgiris, Ooty, Tamil Nadu, 643 001, India

**Keywords:** Dopaminergic neurotransmission, Hyperglycemia, *Mucuna pruriens*, Natural bioactive, Sexual dysfunction, Vajikarana

## Abstract

**Background:**

Dopaminergic neurotransmission is critical to managing a variety of physiological activities, including sexual behavior. Erectile dysfunction is frequently related to low dopamine levels and hyperglycemia, both of which can be alleviated by *Mucuna pruriens*, a natural source of levodopa and other bioactive compounds. The existing hypothesis depicts that decreased dopaminergic neurotransmission and hyperglycemia lead to erectile dysfunction. . .

**Objectives:**

The primary objective of this study was to establish the hypoglycemic action and sexual stimulatory effects of *Mucuna pruriens* in rodents. The secondary objective was to evaluate the effect on general rodent behavior, which can validate the safety profile of *Mucuna pruriens* for clinical use.

**Methods:**

Standardized *Mucuna pruriens* extract was given orally to male rodents for a predetermined amount of time. Blood samples were withdrawn from the male rats to measure the glucose levels at predetermined intervals. During mating trials, sexual activity metrics such as mount frequency and delay, intromission frequency, and ejaculation frequency were recorded. Standard open-field and exploratory behavior tests were used to track general behavior and evaluate any unfavorable or unusual changes.

**Results:**

*Mucuna pruriens* significantly decreased blood glucose levels and increased male sexual activity and behaviors in rodents (mount frequency and latency, intromission frequency, and ejaculation frequency).

**Conclusion:**

Thus, *Mucuna pruriens* can be the alternative natural bioactive to prevent and treat sexual dysfunction.

## 1 Introduction

Sexuality is an important physiological facet of human health that can positively impact the overall mental and functional well-being of humans [1]. Currently, sexual dysfunctions are highly prevalent in both sexes universally. However, due to environmental factors, nutritional deficits, exposure to substances of abuse, neurochemical & endocrine alterations, and comorbidities (hypertension, hyperglycemia), it is alarmingly increasing in males. Male sexual dysfunction is characterized by a series of conditions that notably include Erectile Dysfunction (ED), Premature Ejaculation (PE), and Peyronie's Disease (PD). The prevalence and incidence of sexual dysfunction in males increases with age, with more than fifty percent of men aged above 40 years getting affected globally each year [2]. This seeks immediate non-pharmacological and pharmacological preventative and beneficial strategies as part of the general focus on the quality of life of the rapidly aging populations [3]. Epidemiological studies conducted conclude that approximately fifteen percent of men are affected globally with sexual dysfunction each year [4], and over 150 million men were estimated to have been affected, which was projected to rise to more than 320 million by the year 2025 [5]. Erectile dysfunction (ED) is a common sexual dysfunction in men, which is characterized as the inability to achieve and maintain an erection sufficient to permit satisfactory sexual performance [6]. Due to its very high prevalence, ED has been depicted as a notable public health problem by a National Institutes of Health (NIH) consensus panel [7]. ED may result from an alteration of neurological, psychological, endocrine, and vascular functions, cavernosal impairment, or from a combination of these endogenous and/or exogenous factors. At present, available therapeutic interventions include various pharmacological approaches (endocrine supplementation-testosterone, intra-cavernosal injection-Alprostadil, transurethral therapy-intraurethral pellets, oral medications-phosphodiesterase-V inhibitors), surgery, and vacuum therapy [8,9]. Currently, the existing pharmacological interventions do not treat the current pathology completely; instead can induce iatrogenesis and severe adverse drug reactions. Sildenafil citrate (oral phosphodiesterase-V Inhibitor) is a widely used and successful pharmacological strategy that directly targets the penile mechanism of erection by modifying the hemodynamics in the penis [9]. However, the treatment leads to various iatrogenic and adverse effects, which were frequently reported, such as headache, hypotension, flushing, dyspepsia, nasal congestion, and abnormal vision [10]. Similarly, sildenafil treatment is of great concern to many, as it is contraindicated in several cardiovascular pathologies [9,10]. Thus, there is a great need and demand for newer therapeutic approaches for sexual enhancement and better pharmacological strategies for sexual dysfunction (cost-effectiveness, protection, and minimal adverse drug effects).

Ayurvedic system (ancient Indian medicine) states that several natural bioactives extensively enhance sexual (*Vajikarana*) activity [11,12]. The Ayurvedic natural bioactives with significant sexual activity are *Mucuna pruriens, Pueraria tuberosa, Tinospora cordifolia, Piper cubeba, Boerhaavia difusa, Orchis latifolia, Ficus benghalensis*, and *Ficus religiosa* (Puri, 2003, [13]. *Mucuna pruriens*, commonly known as the velvet bean or Cowhage, is a wild climbing legume that is extensively found in Asia, Africa, and other tropical and sub-tropical regions of the world, including Central and South America [14]. *Mucuna pruriens* belongs to the *Fabaceae* family, *Papilionaceous* sub-family. *Mucuna pruriens* is considered a viable source of dietary supplements due to its valuable bioactives such as alkaloids, proteins (ranges between 23 and 35%), saponins, and amino acids (threonine, proline, tyrosine, phenylalanine, tryptophan, glutamic acid, aspartic acid, serine, lysine, histidine, and arginine) [13]. In addition, Mucuna beans also have high contents of lipids, carbohydrates, fiber, and minerals (potassium, magnesium, calcium, iron, sodium, phosphorus, copper, zinc, and manganese) [15]. Importantly, Mucuna contains key active constituents such as Levodopa (l-DOPA), Serotonin, and 5-Hydroxytryptophan (5-HTP, precursor to serotonin) [16]. l-DOPA (dopamine precursor, 4–7%) in the Mucuna bean makes it an attractive natural alternative bioactive to synthetic dopamine precursors for the treatment of Parkinson's disease [12,17], and other neuropathologies involving dopaminergic denervation in the central and peripheral nervous systems. Besides l-DOPA, *Mucuna pruriens* also contains 5-HTP and Serotonin, another essential precursor and neurotransmitter involved in mood regulation, sleep, and appetite control, and consequently has a positive impact on overall mental health (mood enhancement and stress reduction).

Chronic hyperglycemia can have detrimental effects on various organ systems, including the vascular, nervous, and endocrine systems, all of which play critical roles in male sexual function. Sexual dysfunction in males due to hyperglycemia can be attributed to several interrelated mechanisms, including endothelial dysfunction, vascular impairment, neuropathy or nerve/neuronal damage, hormonal imbalance, formation of advanced glycation end products, reduced nitric oxide synthase activity, and injury to blood vessels. It is important to note that patients with diabetes mellitus or hyperglycemia have an increased risk for sexual dysfunction. Additionally, early and effective management of diabetes mellitus or control of glycemic index can essentially decrease the risk of sexual dysfunction and improve the overall quality of a patient's life.

The Ayurvedic natural bioactive, *Mucuna pruriens,* has been used for centuries for its various pharmacological activities and minimal toxicity. However, there are very few studies that have elucidated the sexual activity of *Mucuna pruriens*. Hence, the current study evaluated the sexual activity of the Ayurvedic natural product, *Mucuna pruriens* extract, using a scientifically accepted animal model with the ultimate purpose of validating the ethnopharmacological actions for the therapeutic treatment of sexual dysfunctions.

## 2 Materials and Methods

*Mucuna pruriens* (MPX) extract was prepared from organically cultivated matured seeds obtained from Zandu Pharmaceutical Works (Mumbai, India, [18]). For the current study, *Mucuna pruriens* seeds were obtained from a single harvest to avoid any variation and to maintain the consistency of the natural bioactives. The MPX formulation was stored in a sealed, airtight container and stored in a cool, dry, dark area. MPX formulation was dispersed in sterile water, shaken thoroughly, and centrifuged, and the supernatant was used for the current experiments.

### 2.1 Animals

Adult male rats display an ordered physiological sequence of motor actions prior to their sexual activities in the presence of a sexually receptive female. Thus, sexual behavior studies of male rats are highly suitable for the analysis and screening of various synthetic drugs or natural bioactives with sexual-enhancing properties. The current study used adult male rats to evaluate the sexual activity of *Mucuna Pruriens* (MPX) extract. Adult male and female (ovariectomized) Sprague-Dawley rats weighing 250-300 gm were purchased from Charles River Laboratories (Wilmington, MA). Male rats were tested for their sexual potency by exposing them to females. The ovariectomized female rats were brought to estrus condition by administering estradiol benzoate (about 52 h) and progesterone (about 4 h) before being introduced to males for the evaluation of sexual activity. Both estradiol benzoate (20 μg) and progesterone (1 mg) were dissolved in olive oil and injected subcutaneously. The controls were administered with olive oil. The animals were selected for experiments in a random manner. The female rats were used repeatedly with an interval of a minimum of one week between each exposure. Although one female was tested against one male, additional females in the estrus cycle were maintained as reserves. All rats were maintained on a reversed light cycle, with free access to food and water. MPX was treated in male rats for three days (500 mg/kg) and fourteen days (250 mg and 500 mg/kg), respectively, and the exhibited general and sexual behaviors (after the introduction of females) were carefully monitored. MPX was dissolved in sterile water and administered intraperitoneally to male rats (each group n = 6). The sterile water-injected group (n = 6) was the control group. The sexual behavioral tests were conducted 1 h after the last injection. Blood samples were withdrawn from the male rats to measure the glucose levels. Recommendations and guidelines for the care and use of the animals in compliance with the NIH Guide for the Care and Use of Laboratory Animals and all the protocols approved by the Scott and White Hospital/Texas A & M, Temple, TX (SW-2004) Institution's Animal Care and Use Committee were followed.

### 2.2 Sexual behavior screening tests

Male and female rats were brought to the observation room and were left there for 10 min or more before beginning the study. One male rat per test chamber was used, and the test started about 5 min later, with the introduction of a receptive female in each test chamber. The treatment of steroid hormones estradiol and progesterone in ovariectomized female rats induced the estrus cycle and resulted in sexual receptivity. Usually, the female displays several proceptive behaviors soon after the introduction to males. These behaviors include ear-wiggling (rapid anteroposterior vibrations of the ears), darting (a short run where the female abruptly stops presenting her posterior to the male) and hopping (a short jump with stiff legs followed by immobility and presentation). Various aspects of sexual activities such as mount frequency (mount frequency is defined as the total number of mounts in 30 min) and latency (mount latency is defined as the time taken by the male to mount the female for the first time), intromission frequency (intromission frequency is defined as the total number of intromissions in 30 min) and latency (intromission latency is defined as the time taken by the male to intromit the female for the first time), and ejaculation frequency (ejaculation frequency is defined as total number of ejaculations in 30 min) were observed and recorded in the male rats. If a female was found not receptive, they were immediately replaced with another female. The sexual behavior of the control and MPX-administered rats was assessed in a quiet room lit with dim white light.

#### 2.2.1 Assessment of the effect of *Mucuna pruriens* on common rodent behavior

The Common Rodent behavioral activities were monitored and assessed repeatedly. Behavioral assessment in rodents was performed regularly following every treatment [19].

### 2.3 *In-silico* computational analysis

Ligands with acceptable pharmacokinetic profiles (absorption, distribution, metabolism, and elimination-ADME) are the fundamental markers/features for an endogenous/exogenous ligand to be an effective drug molecule to diagnose, prevent or treat a disease state. The current study employed the SwissADME tool to assess the pharmacokinetic and pharmacodynamic properties of levodopa and 6,7-dimethoxy-1,2,3,4-tetrahydroisoquinoline-3-carboxylic acid [19].

### 2.4 Statistical analysis

Results are expressed as the average/mean ± SEM. The statistical values of *p* ≤ 0.05 were considered significant. Statistical analyses and data were assessed using one-way analysis of variance (ANOVA) followed by Tukey's Test. Statistical evaluation was assessed using Prism-V software (La Jolla, CA, USA).

## 3 Results

### 3.1 *In-silico* computational analysis

Both the bioactives levodopa and 6,7-dimethoxy-1,2,3,4-tetrahydroisoquinoline-3-carboxylic acid of *Mucuna prurines* are predominant [20], levodopa is considered to be the major bioactive of the *M.prurines*; and the other natural bioactive, 6,7-dimethoxy-1,2,3,4-tetrahydroisoquinoline-3-carboxylic acid isolated from *Mucuna pruriens*, traditionally used for the treatment of Parkinson's disease, and synthesized from l-DOPA, behaves as a peripheral catechol-*O*-methyltransferase inhibitor. Both the bioactives are majorly shown in the same ADME profile (Table 1), through which the compounds exhibit their similar pharmacological effects.

**Table 1:** *In-silico* evaluation of ADME parameters of Levo-Dopa and Isoquinoline alkaloid by SwissADME

		Levo-Dopa	6,7-dimethoxy-1,2,3,4-tetrahydroisoquinoline-3-carboxylic acid
**Physicochemical properties**	Molecular Formula	C_9_H_11_NO_4_	C_12_H_15_NO_4_
	Molecular weight	197.19 g/mol	237.25 g/mol
	Num. heavy atoms	14	17
	Num. aromatic heavy atoms	6	6
	Fraction Csp3	0.22	0.42
	Num. rotatable bonds	3	3
	Num. H-bond acceptors	5	5
	Num. H-bond donors	4	2
	Molar Refractivity	49.55	65.35
	TPSA	103.78 Å^2^	67.79 Å^2^
**Lipophilicity**	Log Po/w (iLOGP)	0.72	1.84
	Log Po/w(XLOGP3)	−2.74	−1.38
	Log Po/w(WLOGP)	0.05	0.27
	Log Po/W(Mlogp)	−2.26	−1.77
	Log Po/w(SILICOS-IT)	−0.07	1.37
	Consensus Log P o/w	−0.86	0.07
**Water solubility**	Log S(ESOL)	0.54	−0.50
	Solubility	6.91e+02 mg/ml ; 3.50e+00 mol/l	7.42e+01 mg/ml ; 3.13e-02 mol/l
	Class	Highly soluble	Very soluble
	Log S (Ali)	1.11	0.46
	Solubility	2.56e+03 mg/ml ; 1.30e+01 mol/l	6.80e+02 mg/ml ; 2.87e-01 mol/l
	Class	Highly soluble	Very soluble
	LogS (SILICOS-IT)	−0.72	−2.69
	Solubility	3.76e+01 mg/ml ; 1.91e-01 mol/l	4.90e-01 mg/ml ; 2.07e-03 mol/l
	Class	Soluble	Soluble
**Pharmacokinetics**	GI absorption	High	High
	BBB permeant	Yes	Yes
	P-gp substrate	No	No
	CYP1A2 inhibitor	No	No
	CYP2C19 inhibitor	No	No
	CYP2C9 inhibitor	No	No
	CYP2D6 inhibitor	No	No
	CYP3A4 inhibitor	No	No
	log Kp (cm/s)	−9.45 cm/s	−8.73 cm/s
**Drug-likeness**	Lipinski	Yes; 0 violation	Yes; 0 violation
	Ghose	Yes; 0 violation	Yes; 0 violation
	Veber	Yes; 0 violation	Yes; 0 violation
	Egan	Yes; 0 violation	Yes; 0 violation
	Muegge	No; 2 violations: MW < 200, XLOGP3<−2	Yes; 0 violation
	Bioavailability Score	0.55	0.55
**Medicinal chemistry**	PAINS	1 alert: catechol_A	0 alert
	Brenk	1 alert: catechol_A	0 alert
	Lead-likeness	No; 1 violation: MW < 250	Yes
	Synthetic accessibility	1.81	2.4

### 3.2 General behavior

The control group, MPX 500 mg/kg (3 days) group, MPX 250 mg/kg (14 days), and MPX 500 mg/kg (14 days) group were monitored regularly for several general behavioral parameters (Table 2); animals were monitored and observed for behavioral using a safety protocol. There was no significant change in the general behavior observed in MPX-treated male rats as compared to the control group.

**Table 2:** Behavioral parameters of male rats after MPX treatment.

Behavioral Parameters	Control	MPX 500 mg/kg (3 days)	MPX 250 mg/kg (14 days)	MPX 500 mg/kg (14 days)
Abnormal posture (head press)	N	N	N	N
Allergic reaction (HSR)	N	N	N	N
Anaphylaxis	N	N	N	N
Eye Redness	N	N	N	N
Mortality	N	N	N	N
Seizure	N	N	N	N
Tremor	N	N	N	N
Tumor	N	N	N	N

✓ ‘Y’ = ‘Yes observed’.

✓ ‘N’ = ‘Not observed’.

✓ ‘HSR’ = ‘Hypersensitivity Reaction’.

Administration of MPX (500 mg/kg) for 14 days significantly increased the mounting frequency (Fig. 1a, n = 6, ∗*p* < 0.0005). MPX (250 mg/kg and 500 mg/kg) administration for 14 days increased the intromission frequency significantly in the male rats as compared to the control (Fig. 2a, n = 6, ∗*p* < 0.05). However, MPX (250 mg/kg and 500 mg/kg) administration significantly lowered the mount latency as compared to the control (Fig. 1b, n = 6, ∗*p* < 0.005). Interestingly, both doses of MPX at both time points had no significant effect on the intermission latency in male rats (Fig. 2b, n = 6). Long-term treatment (14 days) significantly increased the ejaculation frequency as compared to control (Fig. 3, n = 6, ∗*p* < 0.0005). On the third day of this current study, MPX (250 mg/kg) administration significantly reduced the blood glucose levels as compared to the control. The blood sugar levels for control 94.3 ± 1.59 mg/dl and MPX 81.6 ± 1.25∗ mg/dl were noted. The results are expressed as Average (mg/dl), Mean ± SEM (∗p < 0.05, n = 6).

**Fig. 1a:**
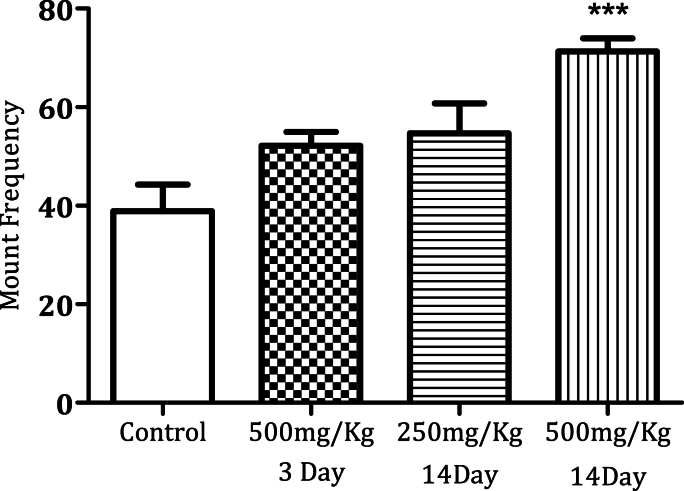
Effect of MPX treatment on mount frequency in male rats. ∗∗∗ Shows dose and time-dependent significant increase in the number of mountings (Control v/s 500 mg/kg, ∗∗∗*p* < 0.0005, n = 6) as compared to the control group.

**Fig. 1b:**
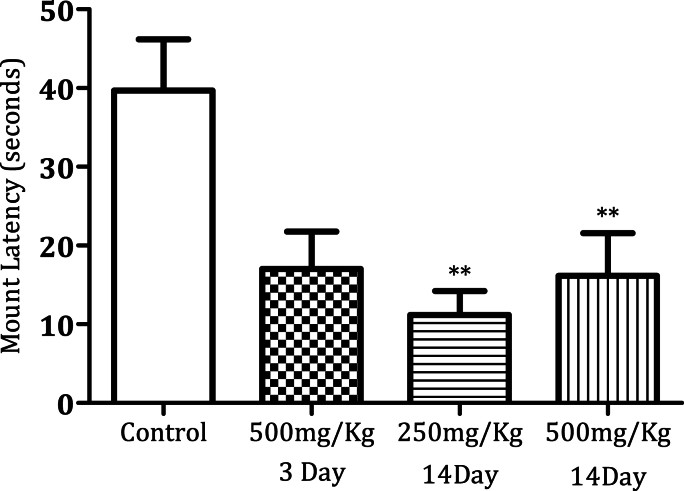
Effect of MPX treatment on mount latency. ∗∗ Shows significant decrease (∗∗*p* < 0.005, n = 6) as compared to the control group.

**Fig. 2a:**
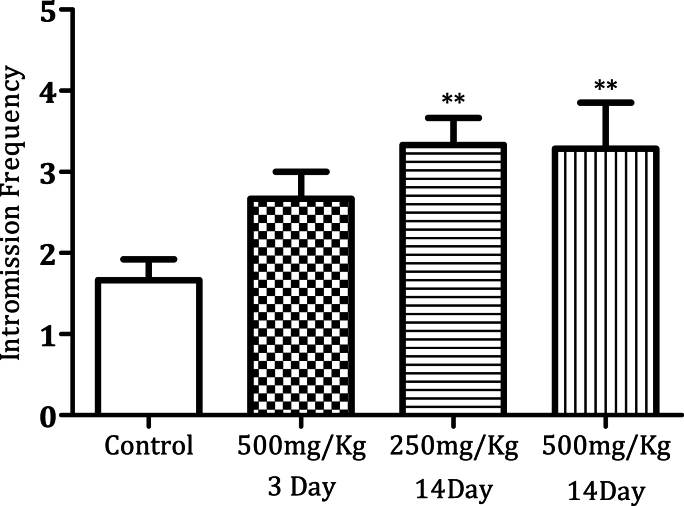
Effect of MPX treatment on intromission frequency in male rats. ∗∗ Shows dose and time dependent significant increase (∗∗*p* < 0.005, n = 6) as compared to the control group.

**Fig. 2b:**
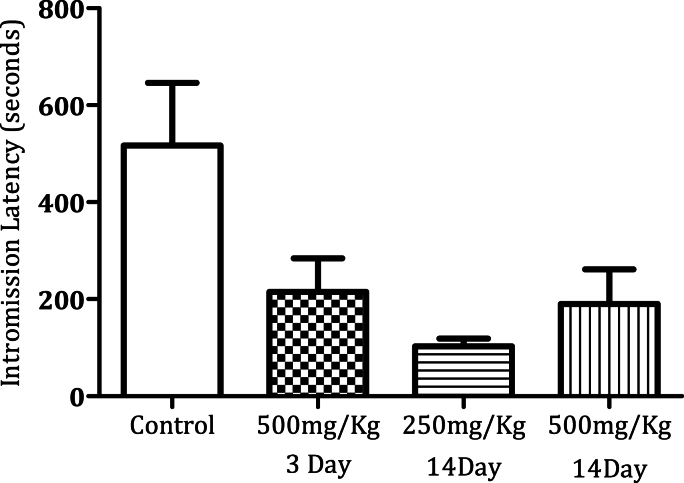
Effect of MPX treatment on intromission latency in male rats. There is no statistically significant difference between MPX treated groups as compared to the control group.

**Fig. 3:**
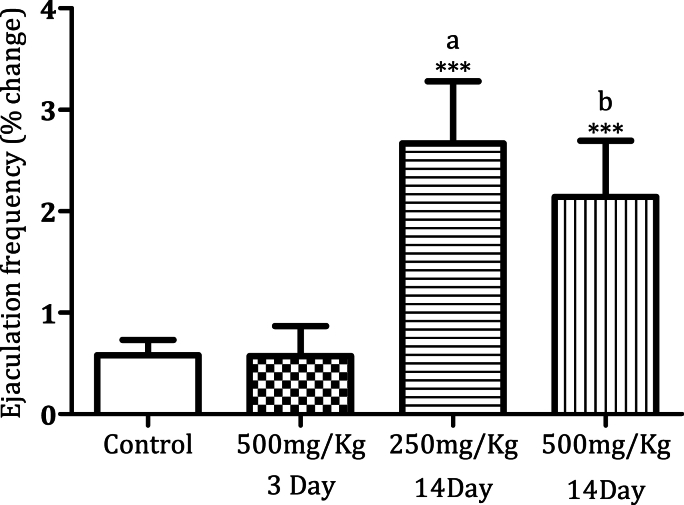
Effect of MPX treatment on ejaculation frequency in male rats. ∗∗∗ Shows significant increase (P = <0.0005, n = 6) as compared to the control group. a: MPX 500 mg/kg (3 days) v/s MPX (250 mg/kg) treatment for 14 days b: MPX 500 mg/kg (3 days) v/s MPX (500 mg/kg) treatment for 14 days.

## 4 Discussion

Ayurveda, an Indian system of traditional medicine, is a well-proven and accepted scientific approach to enhancing the overall well-being of humans globally. This holistic herbal remedy is a scientific approach that has been used to prevent and cure various central and peripheral pathologies by modulating the physiological actions and positively affecting the body's balance, perception, and belief through proper lifestyle. The National Institute of Health (NIH) reports that a substantial American adult population consumes Ayurvedic medicine and this data clearly validates the popularity as patients search for alternatives to the current synthetic medicine [21]. *Mucuna pruriens,* a widely used herbal medicine in *Ayurveda,* has been shown to exert an aphrodisiac, antioxidant, anti-lipid, antitumor/chemotherapeutic, anti-hyperglycemic, antimicrobial (antibacterial), antiprotozoal, anti-snake venom, anti-epileptic, analgesic, and anti-inflammatory effects. The use of Mucuna has been dated and is recorded in Indian history from 1500 BC.

Penile erection is a complex behavioral response that is dependent on the interaction of several diverse humoral and neural events at various levels of the neuroaxis. Dopamine effectively controls movement (through the dopaminergic nigrostriatal pathway), mood, emotion, pleasurable reward, motivation (mesocortical & mesolimbic tract), sexual functions (incertohypothalamic pathway), emesis/nausea (chemoreceptor trigger zone), endocrine secretion (tuberoinfundibular pathway, inhibit prolactin), regulates glucose metabolism (control glucose uptake and metabolism on insulin-sensitive tissues), gastrointestinal motility (peripheral dopamine in the gastrointestinal tract), and immune function. Decades of pathological research have linked a connection between glycemic control and the involvement of the dopaminergic system associated with sexual function [22]. Dopamine plays a critical role in male erectile function, which possesses the ability to achieve and maintain sexual activity. Dopamine (along with testosterone, serotonin, oxytocin, psychological aspects, and environmental factors) regulates a cascade of events in the erectile process, from the initial arousal to the physiological responses required [23]. Giuliano and Allard evaluated the role of dopamine in sexual function in rodents and concluded that the release of dopamine in the nucleus accumbens is positively implicated in the pre-copulatory phase in male rats. The study also established that there is a permissive role in the copulatory or consummatory phase for dopamine released at the level of the median pre-optic area within the hypothalamus. Due to its role in the control of locomotor activity, the integrity of the nigrostriatal dopaminergic pathway is also essential for the display of copulatory behavior [22]. Similarly, the current literature available also depicts the effect of decreased dopamine levels on sperm health and production through its direct and indirect influence on the endocrine system, thereby affecting hormone regulation, which can potentially impact sperm production and quality [24,25].

Interestingly, the *Mucuna pruriens* has a long history of traditional use in Ayurvedic and traditional medicine systems in India for various purposes, including as an aphrodisiac (to treat male infertility) to support overall well-being [16]. Though the therapeutic potential of *Mucuna pruriens* in sexual dysfunction in males has been a subject of interest and research in recent years, more research is needed to clearly establish its efficacy and safety in treating sexual dysfunction. This interest in treating male sexual dysfunction arises from the *Mucuna pruriens* significant content of l-dopa, an amino acid precursor to dopamine, which is closely linked to mood, pleasure, and sexual function. Our previous studies have clearly shown that *Mucuna pruriens* can significantly increase dopaminergic neurotransmission [12,17]. Therefore, a significant increase in the dopaminergic neurotransmission can possibly lead to increased sexual functions and additionally decrease any possible pathologies and increased blood glucose levels leading to sexual dysfunction.

## 5 Conclusion

The findings of the current study summarize the therapeutic potential of *Mucuna pruriens* extract to enhance sexual activity and improve certain aspects of sexual functions in the studied rodents, possibly by treating the possible mechanisms underlying sexual impairment in males. Hence, further research might enlighten new therapeutic approaches that are promising, cost-effective, and associated with minimal adverse effects and iatrogenesis in the treatment of male sexual dysfunction with the traditional natural bioactive *velvet bean “Mucuna."*

## Author contributions

M.D. and B.T.: Performed the experiments, designing, analyzing, visualizing, supervising and interpreting the data; J.B.J., S.P., K.L., R.N. and T.M.: Screening, analyzing and interpreting the data; All authors were involved in drafting and revising the manuscript.

## Declaration of generative AI in scientific writing

All the authors declare no use of AI in scientific writing.

## Sources of funding

Department of Drug Discovery and Development, Harrison College of Pharmacy.

## Conflict of interest

The authors declare the following financial interests/personal relationships which may be considered as potential competing interests: Muralikrishnan Dhanasekaran reports financial support, administrative support, equipment, drugs, or supplies, and statistical analysis were provided by the Department of Drug 10.13039/100002806Discovery and Development, Harrison College of Pharmacy. Other authors declare that they have no known competing financial interests or personal relationships that could have appeared to influence the work reported in this paper.
